# Mapping-by-sequencing accelerates forward genetics in barley

**DOI:** 10.1186/gb-2014-15-6-r78

**Published:** 2014-06-10

**Authors:** Martin Mascher, Matthias Jost, Joel-Elias Kuon, Axel Himmelbach, Axel Aßfalg, Sebastian Beier, Uwe Scholz, Andreas Graner, Nils Stein

**Affiliations:** 1 Leibniz Institute of Plant Genetics and Crop Plant Research (IPK), OT Gatersleben, Corrensstraße 3, 06466 Stadt Seeland, Germany

## Abstract

Mapping-by-sequencing has emerged as a powerful technique for genetic mapping in several plant and animal species. As this resequencing-based method requires a reference genome, its application to complex plant genomes with incomplete and fragmented sequence resources remains challenging. We perform exome sequencing of phenotypic bulks of a mapping population of barley segregating for a mutant phenotype that increases the rate of leaf initiation. Read depth analysis identifies a candidate gene, which is confirmed by the analysis of independent mutant alleles. Our method illustrates how the genomic resources of barley together with exome resequencing can underpin mapping-by-sequencing.

## Background

The recent profound transformation of molecular biology by next-generation sequencing (NGS) technologies [1] and the ready availability of reference genome sequences [2] has enriched the plant geneticist’s toolbox with what Schneeberger and Weigel named ‘fast-forward genetics’ [3]. Combining classical bulked-segregant analysis [4] with aligning NGS read data to a reference genome has made gene cloning essentially a single-step computational procedure once a mapping population has been established [5]. Within a few days’ time, mapping intervals can be delineated *in silico* and mined for likely candidate genes, deprecating marker saturation, and physical mapping of the target interval. Since its original implementation as ShoreMap in an F_2_ population of *Arabidopsis thaliana*, mapping-by-sequencing has been extended to other population types such as isogenic backcross populations [6,7] as well as to other plant and animal species such as rice [8], maize [9], mouse, and zebrafish [10].

All successful attempts at mapping-by-sequencing in these species could take advantage of high-quality map-based reference sequences. A reference genome embeds almost all genes of a species in a genomic context, a crucial prerequisite for mapping-by-sequencing, as sequencing of phenotypic bulks provides only allele frequencies at variant positions, but no genotypic data that could be used to construct a genetic map *de novo* to infer marker order. How this order can be derived in the absence of a reference genome and how rapid NGS-based gene isolation may be implemented in species for which only draft genome assemblies are available is not obvious. Galvao *et al.*[11] have proposed the collinear gene order in related species as a proxy for gene order in species without a reference genomes, but have also noted that this synteny-based approach may adversely affect mapping resolution. A novel bioinformatical procedure to find causal mutations by whole genome sequencing without using positional information has been applied to find causal variants in plant species with small genomes [12].

In addition to its importance for agriculture, barley (*Hordeum vulgare* L.) has been a model organism of genetics throughout the 20th century and boasts excellent resources for forward genetics. A large number of barley mutants had been created from the 1940s to the 1970s when mutation breeding programs flourished [13-16]. These mutant lines have been classified phenotypically and are nowadays maintained and distributed by seed banks. To further support the utilization of these resources in research and breeding, 881 original mutants have been backcrossed to cultivar (cv.) Bowman as a recurrent parent to obtain mutant alleles in a nearly isogenic background. Array-based genotyping of these introgression lines confirmed and broadly delimited introgression intervals [17]. This legacy of half a century of meticulous research has been recently complemented by several mutant populations [18,19] that were obtained in a systemic way via mutagenesis with ethyl methanesulfonate (EMS) to empower reverse genetics.

In this regard, the mutants of barley have been instrumental in confirming candidate genes discovered through mapping in bi-parental populations [20] or association panels [21]. However, the full exploitation of the allelic diversity captured in these resources for basic research and crop improvement has been impeded by the lack of a reference genome sequence of barley. The major obstacles in assembling the barley genome are its sheer size (5 Gb) and its high content of repetitive DNA (80%), which pose a heavy sequencing load and put a challenge for current assembly algorithms [22]. Boosted by the enormous increase in sequencing throughput, extensive sequence datasets have accumulated recently and have been integrated with a genome-wide physical map and high-density genetic maps [23]. A large fraction of low-copy portion of the barley genome is now represented by contigs of a whole-genome shotgun assembly which are positioned with a resolution of approximately 3 cM [24]. Moreover, an exome capture assay designed on the basis of the annotated sequence assembly has made approximately 60 Mb of mRNA-coding sequence accessible to cost-efficient high-throughput resequencing [25].

To date, the complex sequence framework of barley has not been used as a backbone for mapping-by-sequencing. Though the hopes are high, concerns remain that the fragmentary and incompletely ordered structure of the sequence assembly and the only partial representation of the gene complement may stall fast-forward genetics. Leveraging the physically and genetically anchored sequence assembly, exome sequencing and the extensive mutant collections available to the barley research community, we put mapping-by-sequencing to the test in barley and were able to rapidly identify a gene underlying the many-noded dwarf (*mnd*) phenotype.

## Results

### *mnd* mutant phenotype

**Figure 1:**
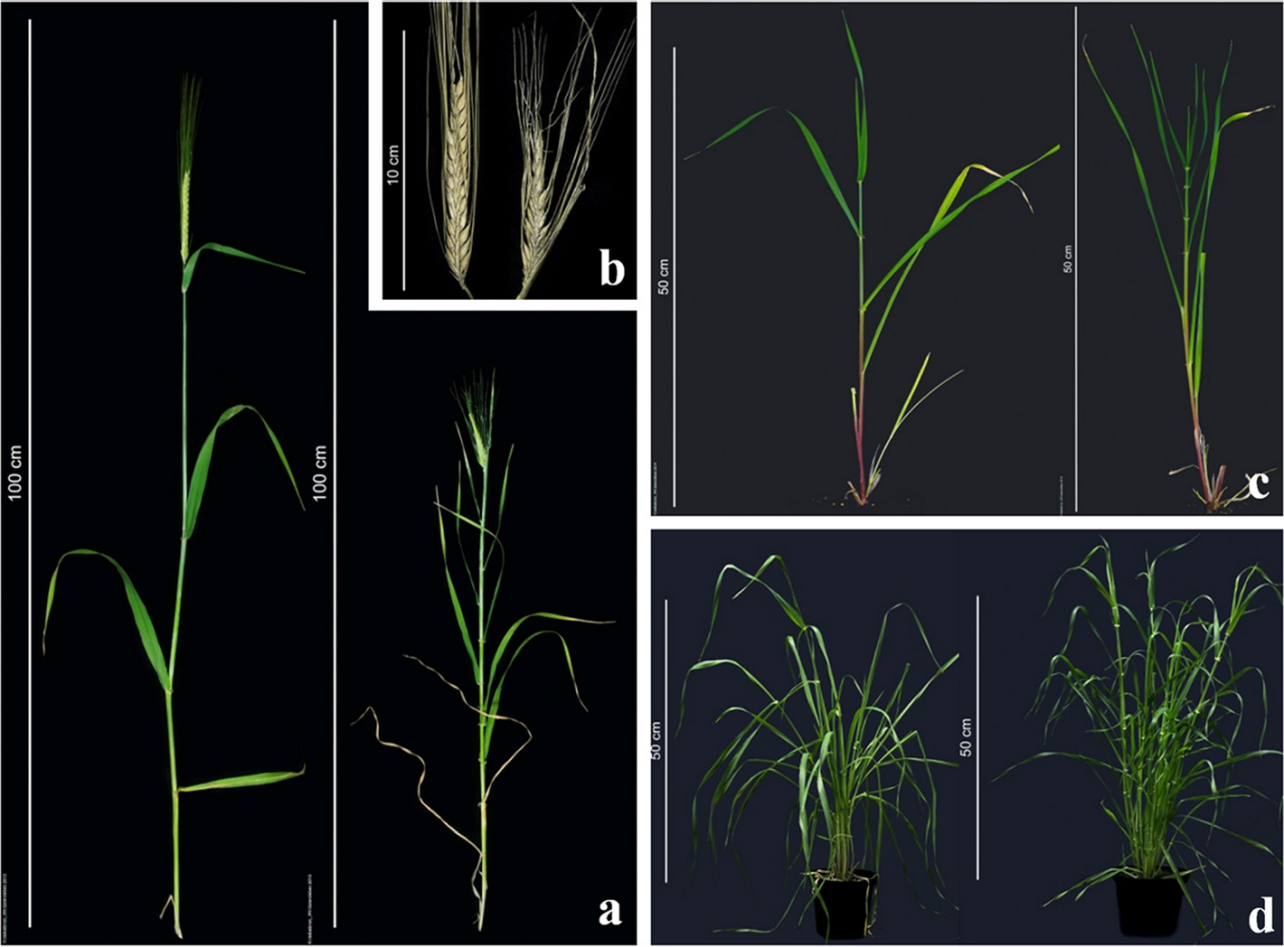
**Phenotypic characteristics of *mnd *plants. (a)** Mutants (right) have a significantly higher number of nodes compared to the wildtype (left) and show a semi-dwarf growth habit. **(b)** Ear length is reduced under field conditions (left: wildtype, right: mutant). **(c)** Leaf formation in early developmental stages is faster in *mnd* plants (right) compared to the wildtype (left). **(d)** Mutant plants (right) grown under greenhouse conditions have more internodes without a dwarfing phenotype. The wildtype is shown to the left.

The original *mnd* mutant was generated by X-ray mutagenesis at our institute in the 1950s [13]. The most conspicuous characteristic of *mnd* plants is their shortened plastochron, that is, a faster rate of leaf initiation. Mutants have on average two times more leaves than wildtype plants as a result of a faster emergence of leaves (Figure 1). Moreover, culm internode lengths are decreased in the mutant. Despite the larger number of internode (eight to nine in the mutant *versus* four to five in the wildtype), plant height is reduced by about one third under field conditions, but not in the greenhouse (Figure 1d). Apart from spacing, also the shape of leaves is altered in the mutant: leaves are narrower and more erect compared to the wildtype. Additional characteristics of *mnd* are an increased number of tillers (vegetative shoot branches arising from lateral meristems) and shorter spikes (Figure 1b; Additional file 1: Figure S1).

### Allele frequency mapping

**Figure 2:**
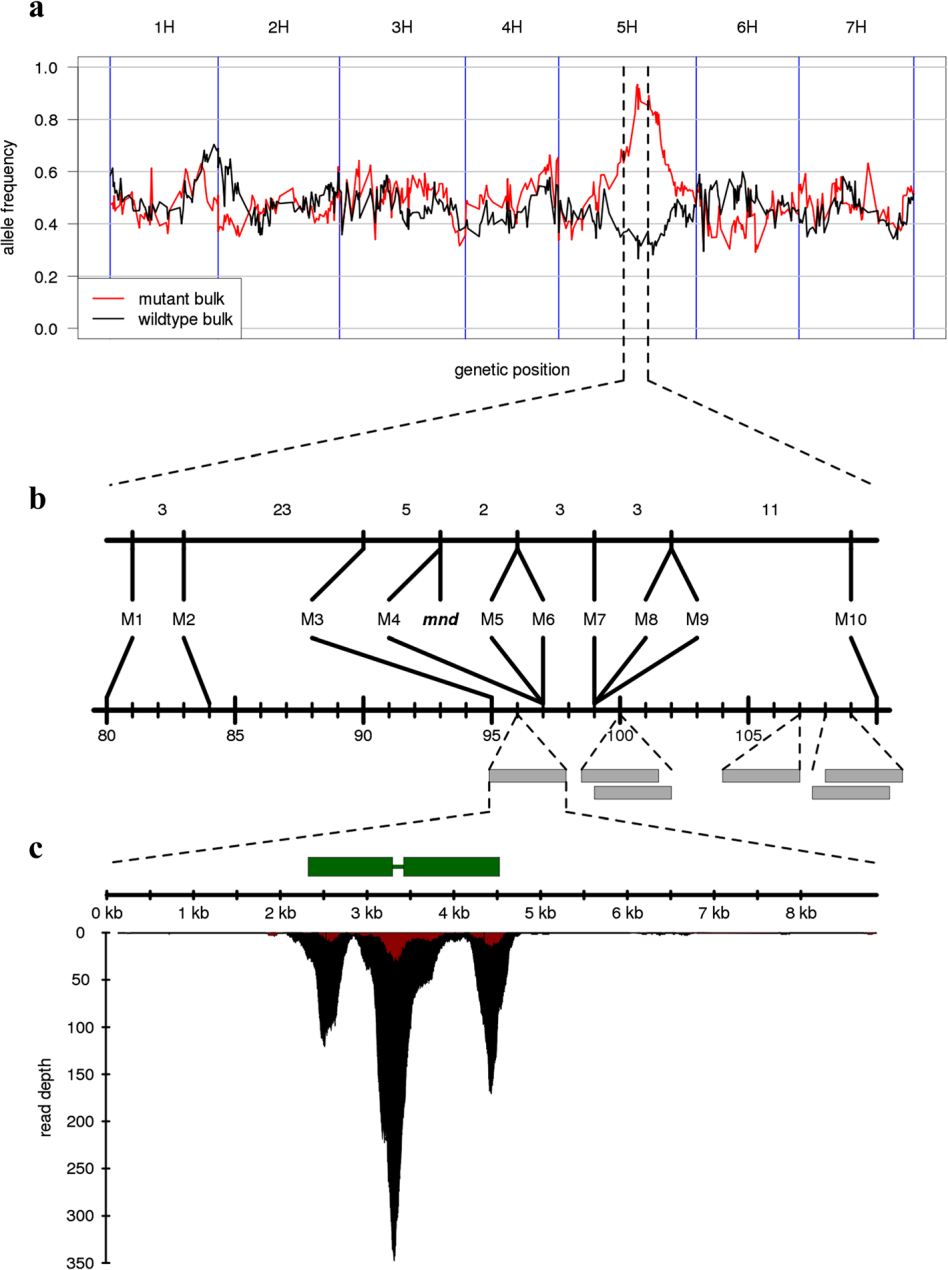
**Mapping-by-sequencing. (a)** The frequency of the alternate allele relative to the Barke reference in the two capture pools is visualized along the integrated physical and genetic map of barley [23]. **(b)** Ten SNPs from the target intervals were converted to CAPS markers and genotyped on the entire F_2_ mapping population. The number of recombinants between the markers (top axis) and marker positions in genetically anchored WGS assembly [24] (bottom axis) are indicated. Sequence contigs carrying large (>150 bp) putative deletions are shown as gray rectangles. **(c)** Read depth of MND (MLOC_64838.2) in the two capture pools. The positions of the two exons of MND in WGS contig 49382 are shown as green rectangles. At the bottom, the number of sequence reads per base position is shown for the mutant pool (red) and the wildtype pool (black). Because of a single heterozygous plant that was erroneously included in the mutant bulk, MND is also present at low read coverage in the mutant pool. Note that the highest coverage peak is in the short intron (130 bp) of MND due to a higher number of redundant capture probes at the ends of the two exons.

We adopted a strategy similar to the ShoreMap [5] and MutMap [8] methods that inspect the genome-wide distribution of allele frequency in phenotypic bulks of an F_2_ population developed by outcrossing the mutant to a wildtype genotype (Additional file 2: Figure S2). Progeny of a cross between an *mnd* plant with a wildtype plant of cultivar (cv.) Barke was selfed to obtain an F_2_ population of 100 individuals. The *mnd* allele segregated in this population as a monogenic recessive trait (19 mutants, 81 wildtype plants, χ^2^ = 1.92, *P* value = 0.17). DNA from 18 mutant plants and 30 randomly selected wildtype plants was combined into two pools, which were subjected to exome capture and subsequent high-throughput sequencing on the Illumina HiSeq2000, yielding 82 million and 70 million 2 × 100 bp read pairs for the mutant and wildtype pools, respectively. Reads were mapped onto the whole-genome shotgun (WGS) assembly of cv. Barke [23] and single nucleotide polymorphisms (SNPs) were detected. The visualization of allele frequencies at SNP positions along the physical and genetic map of barley revealed a single sharp peak on the long arm of chromosome 5H, where the frequency of the mutant allele increased to over 95% and dropped to about 30% in the wildtype pools (Figure 2a). Note that the ratio between the number of plants that are heterozygous at the *mnd* locus and the number of those that are homozygous for the wildtype allele is expected to be 2:1 in the wildtype bulk. Selected SNPs in the interval of 80 to 110 cM in the map of [21] were converted to single marker assays (Additional file 3: Table S2). Genetic mapping in the F_2_ population confirmed these markers to be tightly linked to the *mnd* phenotype (Figure 2b).

### Read depth analysis identifies a likely candidate gene

As X-ray mutagenesis commonly induces large deletions [26], we queried our sequence data for exome capture targets that are covered by sequence reads in the wildtype pool, but not in the mutant pool. As gene models and exome capture targets are given as coordinates on the WGS assembly of cv. Morex, reads were mapped again onto this assembly and read coverage was calculated at each base position and averaged across contiguously covered intervals corresponding to capture targets. Marker assays revealed that we had erroneously included one heterozygous plant in the mutant bulk, which was confirmed by phenotypic analysis of the corresponding F_3_ family. Thus, we expected a small number of sequence reads at the *mnd* locus in the mutant pool originating from the single heterozygote. At genome scale, we identified 435 intervals (whole genome shotgun sequence contigs carrying the respective exome capture targets) that were at least 150 bp and fulfilled our rather relaxed criteria for potential deletions (Additional file 4: Table S3). Of these targets, 18 were mapped by POPSEQ [24] to the broadly defined interval (5H, 80 cM - 110 cM), 278 were mapped to other regions of the genome and 139 were unmapped. Out of all 435 intervals, 48 were located on contigs of the WGS assembly of cv. Morex [23] with high-confidence genes predicted on. All but two of these genes had a functional annotation. Among the contigs carrying putatively deleted capture targets and localized to our target interval, six carried high-confidence genes (Figure 2b, Table 1). One of these, contig 49382 was anchored at 96 cM in the POPSEQ map [24] and thus closest to the allele frequency peak (97%) in the mutant bulk at 97 cM (Additional file 5: Table S1). Moreover, contig 49382 harbored two putatively deleted regions, among them the longest detected interval. Note that a single large deletion would rather show up as several smaller deleted target intervals because exome capture targets only disjoint exons, and introns are represented neither in the mutant nor the wildtype. The deleted regions on contig 49382 overlapped with the two exons of the high-confidence gene MLOC_64838.2 annotated as ‘Cytochrome P450’ (Figure 2c). This gene was the only gene predicted on contig 49382. A BLAST search of the protein sequence against the rice and Arabidopsis genomes identified members of the CYP78A family of cytochrome P450 enzymes. One of these genes, rice CYP78A11, is known as PLASTOCHRON1 (PLA1) [27]. As the rice *pla1* phenotype (rapid leaf initiation, reduced leaf size, and plant height) closely resembles barley *mnd*, we considered MLOC_64838.2 as a promising candidate.

**Table 1:** Deleted target regions within the genetic target interval (5H, 80 cM - 110 cM)

**Morex WGS contig**	**Start position**	**End position**	**Coverage**^**a**^**mutant**	**Coverage**^**a**^**wildtype**	**HC confidence genes**[23]	**Functional annotation**[23]	**Genetic position (POPSEQ)**[24]
contig_49382	2,107	2,455	0.4	24.4	MLOC_64838.2	Cytochrome P450	5H, 96.6 cM
contig_49382	3,871	4,101	1.1	8.8	MLOC_64838.2	Cytochrome P450	5H, 96.6 cM
contig_159829	785	996	0.9	8.2	MLOC_21734.1	CC-NBS-LRR	5H, 99.9 cM
contig_159829	4,501	4,709	0.4	12.4	MLOC_21734.1	CC-NBS-LRR	5H, 99.9 cM
contig_1558349	1,236	1,477	1.5	11.5	MLOC_10070.1	MATE efflux family	5H, 99.9 cM
contig_45126	14,800	14,961	0.8	7.3	AK365660	Tumor susceptibility 101 protein	5H, 107.1 cM
contig_2547452	4,712	4,913	0.1	10.5	AK357178	3’-5’ exoribonuclease CSL4	5H, 108.1 cM
contig_58347	7,926	8,186	1.0	14.5	MLOC_70680.1	DNA repair protein-like	5H, 109.4 cM

^a^Average coverage across the target region.

### Mutant analysis confirms MLOC_64838.2 as *HvMND*

**Figure 3:**
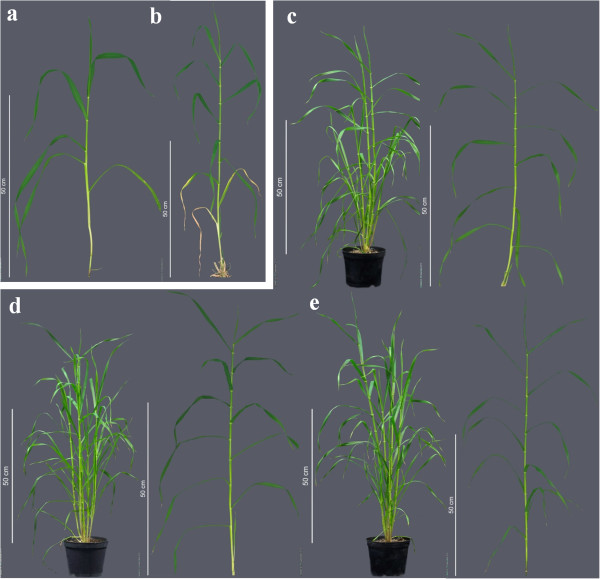
***mnd *mutants.** TILLING mutants **(b)** with a premature stop codon within the MND genes show a significantly faster leaf initiation compared to the wildtype **(a)**. *mnd* mutants in the same genetic background (cv. Kristina) with a single amino acid change **(c)**, a complete gene deletion **(d),** and a premature stop codon **(e)**. The type of mutation did not affect the severity of the *mnd* phenotype under greenhouse conditions. The complete growth stature (left) and a single isolated tiller (right) is shown for each plant in **(c, d, and e)**.

PCR amplification of the candidate succeeded in cultivars Morex and Barke, but failed in the mutant MHOR474. By contrast, we were able to amplify genes that were predicted to be close to MLOC_64838.2 through collinearity to the model grass *Brachypodium distachyon*[28] and were anchored genetically within the mapping interval. Screening of our TILLING (Targeting Local Lesions IN Genomes) population [18] identified 20 EMS mutants with synonymous and 17 mutants with non-synonymous changes. One mutant carrying a SNP (G261A) that led to a premature stop codon in heterozygous state (Table 2) was selected to check the phenotypic effects. Among the offspring of this plant, 15 plants were heterozygous, two were homozygous for the wildtype allele and five were homozygous for the mutant allele. All of the homozygous mutant plants (and only these) showed a significantly increased number of internodes, characteristic of the *mnd* phenotype (Figure 3a,b). Furthermore, introgressions of two Bowman nearly-isogenic lines characterized as *mnd* (BW520 and BW522) had been mapped to chromosome arm 5HL previously [17]. Sanger sequencing of MLOC_64838.2 in BW520 revealed one non-synonymous SNP in the coding sequence. The gene could not be amplified in BW522, whereas all syntenic genes were present (Table 3). We ordered 37 mutant accessions from the Nordic Gene Bank (NordGen) that were described as *mnd.* Resequencing of our candidate in these lines revealed four amino acid changes, 16 premature stop codons, one disruption of a splice site, one 107 bp deletion in the second exon, and six complete deletions (Additional file 6: Table S4). When grown in the greenhouse, all mutants showed the *mnd* phenotype (Figure 3c-e). We considered this large number of molecular lesions found in several independent mutant collections as conclusive evidence that loss-of-function of MLOC_64838.2 underlies the *mnd* phenotype and named this gene as *HvMND*.

**Table 2:** TILLING mutants

**Plant #**	**SNP**^**a**^	**M3 status**	**Effect**^**b**^
13339_1	C160T	Heterozygote	P54S
6869_1	C180T	Homozygote	L60=
4098_1	G261A	Heterozygote	W87*
3895_1	G306A	Homozygote	R102=
4642_1	G435A	Heterozygote	M145I
6631_1	G465A	Heterozygote	R155=
4555_1	G468A	Heterozygote	E156=
9906_1	C609T	Heterozygote	S203=
13573_1	G639A	Homozygote	Q213=
7036_1	C657T	Heterozygote	S219=
16177_1	C671T	Homozygote	A224V
4610_1	G691A	Heterozygote	G231S
3158_1	G699A	Homozygote	E233=
6465_1	G736A	Heterozygote	V246M
9172_1	G755A	Heterozygote	G252D
2983_1	G846A	Homozygote	Q282=
16204_1	C861T	Heterozygote	D287=
13675_1	C864T	Homozygote	H288=
14226_1	G1221A	Heterozygote	D365N
15112_1	C1251T	Homozygote	L375=
10971_1	G1289A	Heterozygote	R387=
11186_1	G1314A	Homozygote	V396M
11295_1	G1319A	Heterozygote	G397=
15186_1	G1332A	Heterozygote	V402I
10869_1	C1365T	Homozygote	L413F
10498_1	C1389T	Homozygote	L421=
8989_1	C1460T	Heterozygote	G444=
10895_1	C1464T	Heterozygote	L446F
4786_1	C1471T	Heterozygote	P448L
15867_1	C1511T	Heterozygote	T461=
7736_1	G1566A	Heterozygote	A480T
13507_1	G1569A	Homozygote	G481R
15012_1	G1642A	Homozygote	S505N
13490_1	G1651A	Heterozygote	G508E
13955_1	C1678T	Heterozygote	A517V
14646_1	C1707T	Heterozygote	L527=
14033_1	C1724T	Homozygote	S532=
10243_1	C1748T	Heterozygote	N540=

^a^Coordinates based on genomic sequence of cv. Barke.

^b^Amino acid changes are given given as positions in protein sequence of cv. Barke. Stop codons are indicated by ‘*’, synonymous changes by ‘=’.

**Table 3:** **Sequence variation of MND in nearly isogenic lines of cv. Bowman **[17]**described as *mnd***

**Name**	**Allele**	**Chr**	**Mutagen**	**Original cultivar**	**Backcross**	**Mutation**	**Effect**
BW516	mnd.f	NA	Spontaneous	UT1713	BC3	/	
BW517	mnd.h	NA	Fast neutron	Steptoe	BC1	/	
BW518	mnd1.a	4HL	Spontaneous	Mesa	BC8	/	
BW519	mnd3.d	3H	Gamma-ray	Montcalm	BC6	/	
BW520	mnd4.e	5HL	Ethyl methanesulfonate	Akashinriki^a^	BC6	G1642A	R505K
BW521	mnd5.g	NA	Spontaneous	Logan/ND15053	BC3	/	
BW522	mnd6.6	5HL	Ethylene imine	Bonus	BC6	Complete deletion	

^a^No reference DNA of Akashinriki was available. We excluded all SNPs that were shared with cv. Morex and Barke.

### MND is a member of the CYP78A subfamily of cytochrome P450 enzymes

**Figure 4:**
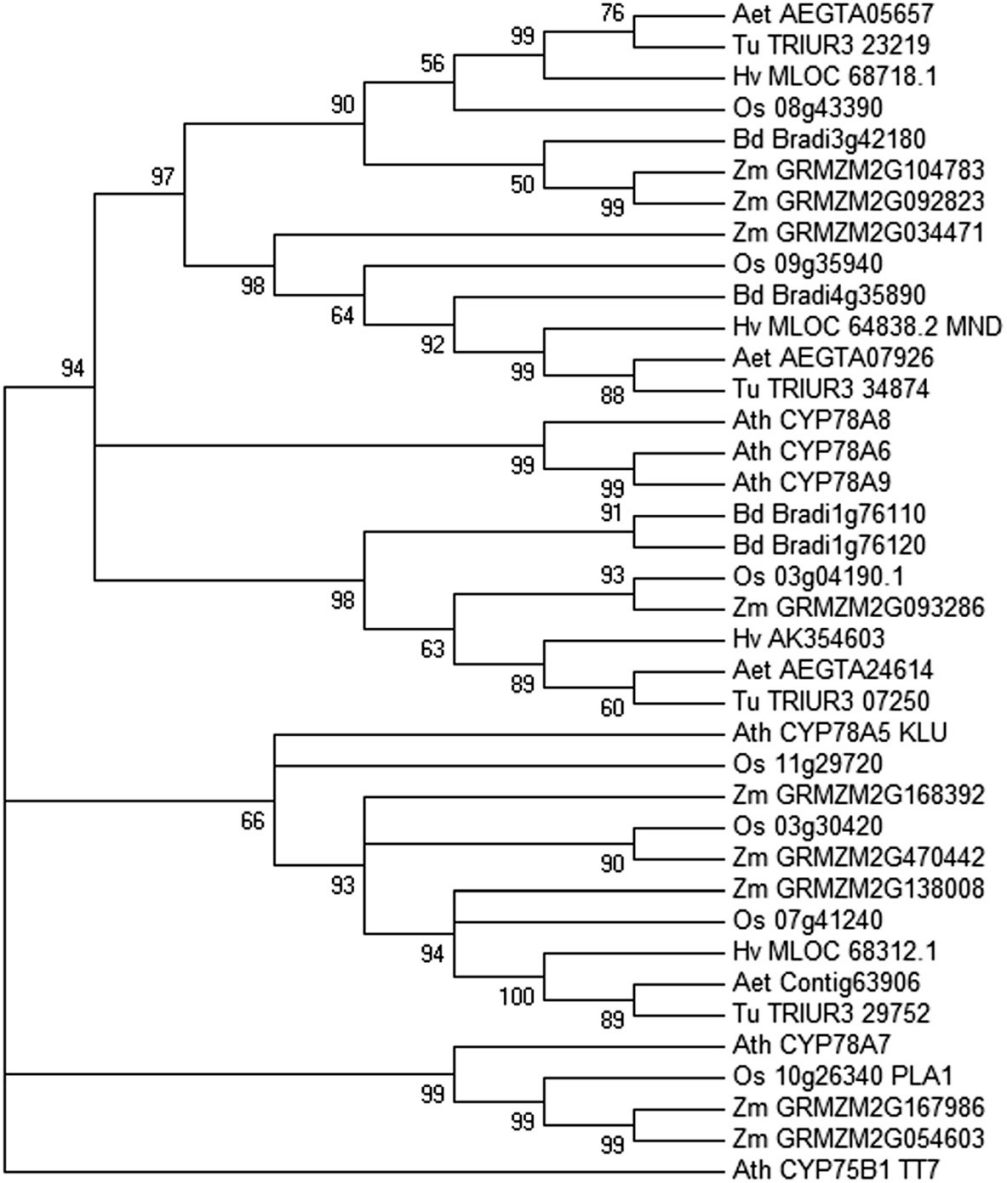
**Phylogenetic analysis of CYP78A genes.** A phylogenetic tree of 38 protein sequences of CYP78A from different species was constructed with MEGA5. Abbreviated species names are given before gene identifiers: *Aegilops tauschii* (Aet), *A. thaliana* (Ath), *B. distachyon* (Bd), *H. vulgare* (Hv), *Oryza sativa* (Os), *T. urartu* (Tu), *Zea mays* (Zm). Gene names are given after identifiers if available. The CYP75B1 gene TT7 of *A. thaliana* was used as an outgroup. The bootstrap method was applied to test for statistical significance of branches. The percentage of replicate trees in which the associated taxa clustered together in the bootstrap test (1,000 replicates) is shown next to the branches. Branches with insufficient bootstrap support (<50%) were collapsed to obtain a consensus tree.

MND is a member of the CYP78A family of cytochrome P450 enzymes. We found four CYP78A genes in the whole genome shotgun assembly of barley (Figure 4). Though the *mnd* phenotype mimics *pla1*, MND is not an ortholog of PLA1. The ortholog of MND in rice, Os09g09g3594, is located in a syntenic region on rice chromosome 9 [28] and shows 75% identity with MND on the protein level. PLA1 does not have a clear ortholog in barley (Figure 4), but has approximately 54% amino acid sequence identity to MND and two other CYP78A genes, MLOC_68312.1 and MLOC_68718.1. As PLA1 has orthologs in maize and Arabidopsis (Figure 3), an ancient ortholog of PLA1 might have been lost in the *Poaceae* lineage after its split from rice and maize. In line with this hypothesis, we did not find PLA1 orthologs in barley, the wheat progenitors, *T. urartu* and *Ae. tauschii*, and *B. distachyon*.

**Figure 5:**
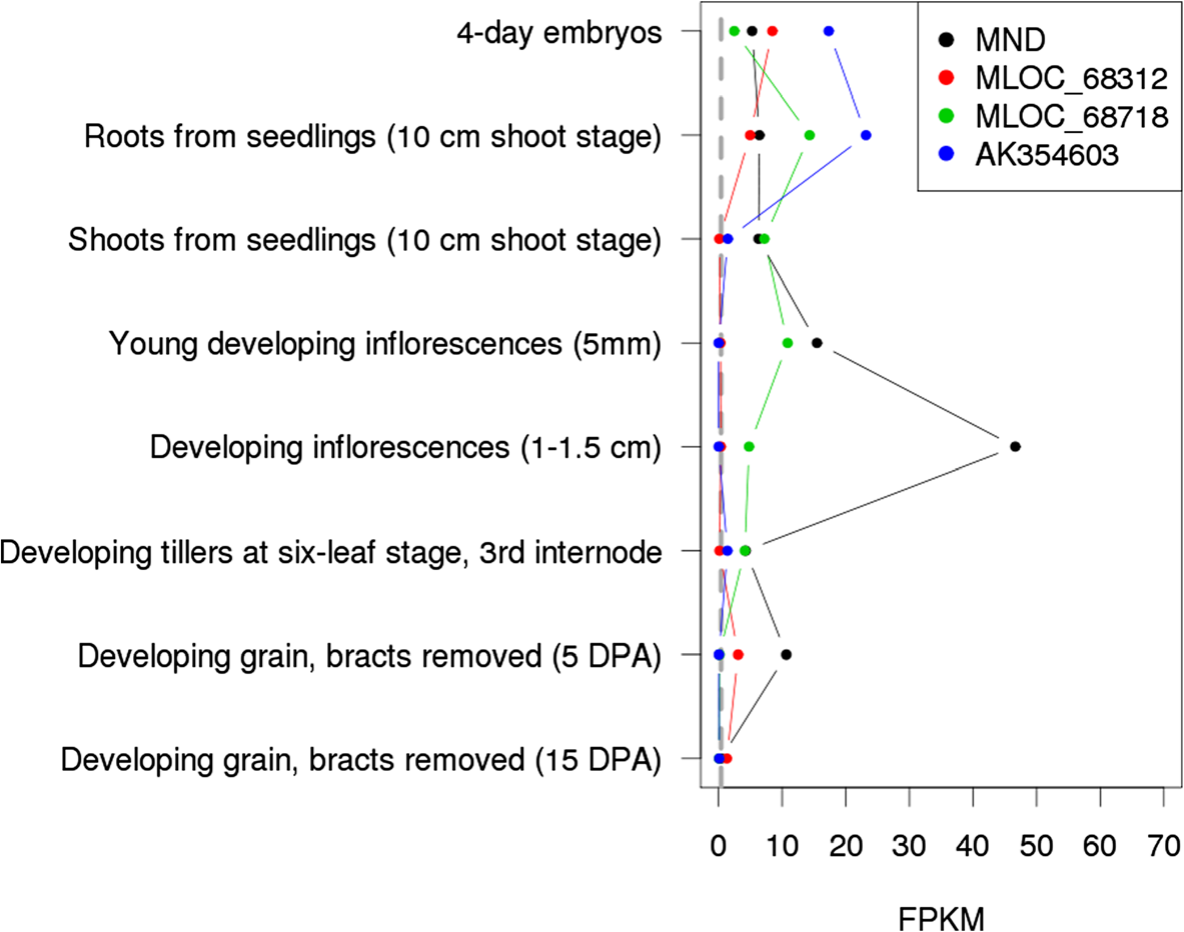
**Expression of *MND *and three other *CYP78A *genes of barley.** Transcript abundance is given as fragments per kilobase of exon per million reads mapped (FPKM) across eight different tissues or developmental stages. A gene was considered expressed if its FPKM value was above the threshold of 0.4 [23] (marked by gray line). All data were taken from [23].

We looked up the expression profile of *HvMND* and other barley genes of CYP78A family in the eight tissues examined by The International Barley Genome Sequencing Consortium [23]. Expression of CYP78A genes was found across all tissues, with different genes of the family being most abundant in different tissues (Figure 5). Among the four CYP78A genes, *HvMND* was the most ubiquitous, being expressed in all samples, although only weak expression was detected in developing grains 15 days after anthesis.

### A physical map of the *mnd* locus

There may be concerns as to the general applicability of our strategy to other map-based cloning projects. The isolation of MND was facilitated by the facts that its homolog PLA1 in the model species rice is well characterized and that the phenotype of PLA1 knockout mutants mirrors *mnd*. If, moreover, MND had not been represented in the exome capture target space, no obvious candidate could have been pinpointed. In this case, the distribution of allele frequency confirmed by genetic mapping of markers developed from *in silico* variants would have only delimited a target interval to be subjected to further scrutiny. As was proposed earlier, the genome-wide physical map of barley should principally obviate the need of constructing local physical maps by map-based cloning to delimit candidate genes [29]. BAC survey sequence data associated with the physical map of barley [23] can be used to associate marker sequences or candidate genes with physical contigs, whose minimum tiling paths [29] can then be sequenced. Thus it was our intention to test whether the information provided by the bulked-segregant sequencing experiment was sufficient to select a physical contig of the genome-wide physical map for delimitation of the target locus region and identification of a candidate gene.

**Figure 6:**
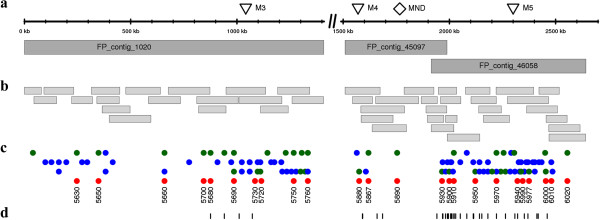
**A physical map of the *mnd *locus. (a)** Fingerprinted (FP) contigs carrying flanking and co-segregating markers (triangles) as well as the MND gene (diamond). The physical map is not contiguous between contigs 1020 and 45097. A scale bar for all panels is given on top. **(b)** Sequenced BACs. BACs were positioned according to their FPC coordinates [23]. **(c)** Gene models and orthologous *Brachypodium* genes. Tracks (from top to bottom) mark the positions of (1) gene models present in both *de novo* predictions with Augustus and the IBSC gene models (green - high-confidence (HC) IBSC genes, blue - low-confidence (LC) IBSC genes); (2) gene models only predicted by Augustus; (3) gene models predicted by IBSC (green - HC genes; blue - LC genes); (4) orthologous *Brachypodium* genes, only the last four digits of the gene identifier Bradi4g3xxxx are given. **(d)** SNPs discovered by exome sequencing and anchored to BAC sequences are marked by vertical lines.

We put this strategy into practice to retrieve the physical map around the MND locus (Figure 6). The major steps towards this aim were the identification of BAC contigs of the barley genome physical map harboring MND as well as its flanking markers, sequencing the minimum tiling paths (MTPs) of these contigs and perform integrative sequence analysis to predict gene models on the BAC sequence assemblies. First, we identified through BLAST searches against the sequence resources integrated to the physical map of barley [23] two fingerprinted contigs, contig_45097 and contig_46058, which harbored two genes whose orthologs in *Brachypodium* were the closest neighbors of the ortholog of MND, as well as the co-segregating and a distal flanking markers M4 and M5. Likewise, contig_1020 was found to harbor marker M3, flanking MND in proximal direction. We found no BAC sequences with high similarity to MND. This is not unexpected as only 1.1 Gb of genomic sequence information (approximately 20% of the barley genome) is directly provided by the physical map of barley (6,278 sequenced BAC clones, BAC end sequences) [23]. However, a BAC harboring MND and assigned to fingerprinted contig_45097 was identified through BAC library screening.

Next, we assembled the MTPs of these three physical contigs (Figure 6a) by sequencing 38 BACs (Figure 6b; Additional file 7: Table S5) on the Illumina HiSeq2000. Single BACs were assembled to ‘phase-1’ quality, that is, unordered contig sequences. All-against-all BLAST searches of BAC assemblies confirmed the contiguity of contigs 46058 and 45097 as well as the overlap between them. Contig_1020 did not overlap with either of them. Markers M4 and M5 were located on a contiguous sequence scaffold, which enabled to us to estimate an approximate ratio between physical and genetic distance at the MND locus of approximately 740 kb per cM.

In the following step, gene models (Figure 6c) were predicted on repeat masked BAC assemblies by using an *ab initio* method and through alignment of gene models defined on the Morex WGS assembly [23]. Overall, 98 non-redundant gene models were defined on the BAC sequences. Twenty-five genes were found by both methods, 35 were only predicted *ab initio* and likely represent pseudogenes. Thirty-eight genes were included only in the IBSC annotation, the majority (23 genes) of them classified as low confidence transcripts, which are also putative pseudogenes or gene fragments. Gene order was largely collinear to *Brachypodium* with some minor rearrangements (Figure 6c). Synteny enabled us to orient contig_1020 relative to the other two contigs.

Finally, we attempted to estimate the size of the gap that was remaining between fingerprinted contigs 1020 and 45097 and to find additional BACs that may bridge it. As 10 *Brachypodium* genes between Bradi4g 35770 and Bradi4g35860 are missing, the gap between contigs 1020 and 45097 may size up to several hundred kilobases, or the gap is small and may represent a region with lack of collinearity between barley and *Brachypodium*. We linked WGS contigs carrying the barley orthologs of the ‘missing’ *Brachypodium* genes to end sequences of BACs that were part of two short physical contigs (45219 and 45903) of sizes 227 and 236 kb (Table 4). These contigs carry the orthologs of Bradi4g35840 and Bradi4g35800, further supporting overall collinearity with *Brachypodium* in this genomic region. Moreover, one BAC end sequence (HF198106) pertaining to contig_45219 matched with high identity (99.9% identity over 755 bp) to two BAC sequences of contigs_45097, indicating that these two FP contigs may overlap.

**Table 4:** **Additional fingerprinted (FP) BAC contigs at the *mnd *** **locus**

	**contig_45219**	**contig_45903**
Genetic position^a^	5H, 97 cM	5H, 96 cM
Length	227 kb	236 kb
BAC	HVVMRXALLeA0202J12	HVVMRXALLhA0201C20
BAC end sequence^b^	HF177908	HF089656
Morex WGS contigs	contig_57438	contig_46356
Barley high-confidence gene	MLOC_70099.11	MLOC_62256.1
Orthologous *Brachypodium* gene	Bradi4g35840	Bradi4g35800

^a^According to [29].

^b^GenBank accession number [23].

In summary, at the genetic resolution provided by 100 F2 plants, we were not able to obtain in one step a single physical sequence scaffold of overlapping BAC clones from the *MND* locus between the two closest flanking markers. However, the remaining gap may be closed by sequencing the MTP of the two additional FP contigs identified based on conserved synteny information to Brachypodium. Furthermore, increasing the genetic resolution significantly to several thousand meioses, as often required in barley, may allow to resolve recombinations between marker M4 and the *MND* gene, which would result in landing with flanking markers on a single BAC contig scaffold provided by the physical map of barley. Thus, in spite of the advanced genomic resources that are now available for barley, an iterative process involving more than one round of MTP sequencing and overlap analysis may still be required to obtain a contiguous physical map of a candidate locus.

## Discussion

We have implemented mapping-by-sequencing in barley. Through sequencing two small phenotypic bulks from an F_2_ mapping population of 100 individuals segregating for the *mnd* phenotype, we were able to identify in a single sequencing experiment the deletion of a cytochrome P450 gene of the CYP78A subfamily as a likely candidate for the causal mutation. Resequencing of this candidate in other *mnd* mutants from several independent sources revealed a partial as well as complete deletion alleles, truncated protein products, splice site mutations and single amino acid substitutions, in summary confirming our candidate as the *MND* gene.

Previous mapping-by-sequencing experiments have mainly targeted EMS mutants. In rice, mapping-by-sequencing has been combined with local *de novo* assembly to clone a resistance gene missing from the reference genome, that is, the mutant harbored an insertion relative to the reference [30]. Our results show that mapping-by-sequencing can also easily be adapted to deletion mutants obtained by X-ray or fast neutron mutagenesis, the major adjustment to the analysis procedure being the inspection of read depth instead of SNP effects on coding sequence. As we mined our sequence data, we prioritized large (≥150 bp) deletions. It may be necessary to relax this criterion as the spectrum of radiation-induced mutations also includes deletions of various sizes and even single base substitutions [31]. Of note, we could make use of an existing WGS assembly of one parent of our mapping population [23]. Otherwise, we would have used the assembly of cv. Morex as a reference for read mapping and sequenced one parent to determine its genomic background relative to Morex, similar to the procedure described in [32]. In the present study, we genotyped the individuals of our mapping population using single-marker assays developed from SNP detected in the exome sequencing data. Although these data confirmed and refined the target interval determined through mapping-by-sequencing, additional genotypic data of a mapping population are in general not necessary supplements to a mapping-by-sequencing experiment. In the present study, even a broadly defined interval of 30 cM (5H, 80 to 110 cM) harbored only six deleted capture targets overlapping with high-confidence genes. Completely forgoing genetic mapping, however, for instance by only comparing read depth in sequencing for one mutant and one wildtype individuals, does not seem advisable as it would be challenging to prioritize candidates without any additional positional information.

A simulation study [33] has recently highlighted pool size, sequencing depth, and recombination frequency as key determinants of mapping resolution in mapping-by-sequencing experiments. As we targeted a deletion mutant located in a highly recombinogenic subtelomeric region, even a small pool of mutant plants selected from a population of 100 plants, delimited a mapping interval small enough to clearly prioritize a single deleted region. By contrast, genes located in the genetic centromeres of barley chromosomes, where meiotic recombination is severely suppressed, are notoriously difficult to clone [34-36] and further research should investigate whether sequencing-based methods can make the rarely recombining regions accessible to positional cloning.

Sequencing depth was difficult to control in our study, as we employed exome capture to reduce the genomic complexity of DNA samples prior to sequencing. For the time being, we consider complexity reduction as a necessary evil to perform cost-efficient resequencing experiments in the large genomes of barley (5 Gb) or related Triticeae such as wheat (17 Gb) and rye (7 Gb). For instance, sequencing both pools to 20× whole genome coverage would have required six lanes of a Hiseq2000, while we used only one for exome sequencing. As the capture target comprises only approximately 60 Mb of the barley gene space and has been estimated to capture approximately 75% of the sequence of high-confidence exons reliably [25], exome sequencing always incurs the risk of missing the target gene (or those parts of its sequence that contain the causal mutation). Even so, the analysis of allele frequency distribution in phenotypic bulks would always afford a sufficient number of markers to delineate genetically a target interval, which may then be analyzed in further details. If, for example, MND had not been in the capture space, we would still have been able to identify BAC contigs with closely flanking and co-segregating markers. Increasing the size of the mapping population may then have further reduced the target interval. We have not made further efforts to close gaps in the physical map between the two closest flanking markers, since the International Barley Genome Sequencing Consortium is currently sequencing the MTP of all chromosomes, so respective sequence assemblies of all BAC contigs will become available in the near future.

Mapping-by-sequencing is robust enough to tolerate some experimental error, as even a single heterozygote in the mutant pool did not prevent us from detecting the deletion of *HvMND*. An alternative to pooled sequencing of phenotypic bulks, which confounds the identity of individual samples, is genotyping-by-sequencing (GBS) of an entire mapping population. GBS couples digestion with restriction enzymes to reduce the complexity of DNA samples with barcoded high-throughput sequencing for cost-effective multiplexed genome-wide genotyping [37,38]. As GBS, in contrast to exome capture, produces only short sequence tags and no contiguous gene sequences, the causal polymorphism is likely to be missed. For instance, absence of GBS tags in genes is no evidence for a deletion, but may simply be caused by the absence of suitable restriction sites. Consequently, GBS would necessitate follow-up experiments before a candidate can be determined with any confidence. For instance, GBS may be supplemented with whole-genome or exome sequencing of the parents of the mapping population to obtain a variation database for the design of single marker assays for further fine-mapping, or the target interval delineated by GBS may be mined for candidate genes based on an educated guess assisted by the information provided by the annotated reference assembly. A better balance between complexity reduction and multiplexing might be achievable with barcoded exome capture of an entire mapping population or selected individuals of phenotypic bulks. However, the number of samples to be processed with a single commercial exome capture kit is currently limited to 24 due to technical restrictions. A possible solution could be to combine deep multiplexing protocols [39] with exome sequencing.

A recapitulatory word of caution may not be amiss at this point. The immediate success of a mapping-by-sequencing experiment, that is, pinpointing a candidate in a single step, can be hindered by many factors. Beyond an intrinsic dependence of genetic mapping on recombination rate and the degree of polymorphism between the parents of the mapping population, sequence-based methods are contingent on genomic resources. In barley, further complexity is added both by incomplete reference sequence information and incomplete resequencing data as a result of complexity reduction and we caution researchers adopting our strategy that they may not meet with success in as straightforward a manner as we did.

In the present study, the identification of a candidate for MND was facilitated by the previous characterization of a homolog in rice and the advantageous ratio between physical and genetic distance at the target locus (<1 Mb per cM). Nevertheless, we believe our result to be a showcase for what mapping-by-sequencing can achieve in the context of the current genomic framework of barley despite of its fragmentary structure. The contigs of the whole genome shotgun assembly serve, as far as read mapping is concerned, as effective surrogates for the pseudomolecules of a high-quality reference genome, because the low-copy portion of the barley gene space is reasonably well represented by them. Physical and genetic maps - occasionally assisted by synteny to the model grasses - localize these contigs with sufficient density and resolution to order the majority of sequence variants discovered through exome capture. The functional gene annotation - though mainly based on sequence similarity - is accurate enough to identify the correct gene family of MND.

MND and its rice homolog PLA1 are part of the CYP78A family of cytochrome P450 enzymes, which have been proposed to generate a novel mobile signaling compound involved in the regulation of organ size and cell proliferation of vegetative and reproductive tissue in plants [40]. The reactions catalyzed by CYP78A genes and the regulatory pathways governing their activity are largely unknown [40]. *In vitro* results indicated that CYP78A enzymes catalyze the hydroxylation of fatty acids [41,42]. Members of the CYP78A family may act in the same physiological pathway as ALTERED MERISTEM PROGRAM 1 (AMP1), a glutamate carboxypeptidase, whose *Arabidopsis* mutants show pleiotropic phenotypes such as a shortened plastochron, aberrant meristem programs, and early flowering [43]. A homolog of AMP1 in rice, PLASTOCHRON3, was also cloned as a plastochron mutant [44]. Whereas both CYP78A and AMP1 mutants of Arabidopsis and rice also exhibit an altered seed size [45-47], we did not see any effect on seed size in *mnd* plants (data not shown).

Phylogenetic analyses have shown that CYP78A enzymes have evolved differently in the Poaceae relative to rice and maize and suggested that MND may have taken over the functions of a lost ortholog of rice PLA1 and *Arabidopsis* CYP78A7. This supports the hypothesis that several CYP78A enzymes act in the same physiological pathway and may catalyze similar biochemical reactions [40]. Resolving the unknowns about the substrate(s) of CYP78A enzymes and their upstream regulators [40] seems an attractive research goal insomuch, as the potentially beneficial effects of these genes on important agricultural traits such as the size of seeds and fruits [47,48], the balance between endosperm and embryo [45] and growth stature [27] might make them valuable breeding targets if adverse effects like increased tillering can be kept to a minimum.

## Conclusions

In conclusion, we have demonstrated the feasibility of mapping-by-sequencing in barley by combining reduced representation sequencing, computational analyses contextualized by comprehensive genomic resources, and mining the extensive mutant collections of barley. Similar approaches may be adopted by other map-based cloning projects in barley and in related species with large genomes, if a comparable genomic infrastructure is available for them.

## Materials and methods

### Plant material and phenotyping

The *mnd* mutant was obtained from the genebank of IPK Gatersleben (accession: MHOR474). This mutant had been induced by X-ray mutagenesis of barley cv. Saale [13]. An F_2_ population was developed by crossing the mutant to cv. Barke. One hundred F_2_ plants were grown to full maturation under greenhouse conditions in 2012 (18°C / 16°C day / night temperature). Natural light as well as additional sodium lamps were used for illumination. Twenty F3 offspring plants of each F_2_ individual were grown in 2013 to corroborate phenotypic scores. One half of the F_3_ plants were grown in pots under greenhouse conditions, the other half were grown in a nursery under field-like conditions. Plants were visually phenotyped for the number of internodes, spike length (five spikes per plant), tiller number and plant height (height of the main tiller). Plants with more than five internodes at full maturity were classified as carriers of the *mnd* allele. Bowman nearly-isogenic lines described as *mnd*[17] were obtained from the James Hutton Institute (Dundee, UK). Additionally, 37 accessions, phenotypically classified as *mnd*, were ordered from the Nordic gene bank (NordGen, Alnarp, Sweden) and cultivated under greenhouse conditions.

### Preparation of genomic DNA

Plant material was harvested of young seedlings at three-leaf stage and DNA was extracted according to a modified cetyl-trimethylammonium bromide-based (CTAB) protocol of [49]. Volumes of reagents were adjusted to 1.2 mL to accommodate a 96-well plate format.

### Exome sequencing

DNA from 18 mutant and 30 wildtype plants was combined into two pools. Exome capture and sequencing was performed according to the protocol of [25].

### Read mapping and allele frequency visualization

Reads (2 × 100 bp) of the mutant and wildtype pools were mapped against the whole-genome shotgun assembly of barley cv. Barke [23] with BWA [50] version 0.6.2 (commands ‘aln’ and ‘sampe’). Single-sample SNP calling was performed for each pool with SAMtools version 0.1.18 [51]. Allele frequencies in both pools were calculated as the number of reads supporting the mutant allele divided by the number of reads at a SNP positions with a custom AWK script (Additional file 8: Text S1) and visualized along the integrated physical and genetic map of barley [23] using standard functions of the R statistical environment [52]. For visualization, allele frequencies at SNP positions with at least 30-fold coverage in both pools were averaged in 1 cM bins. SNPs with allele frequencies ≥80% in both pools were not considered. Only bins with at least 30 SNPs were considered. The genetic positions of sequence contigs of cv. Barke were downloaded from MIPS PlantsDB [53,54].

### Read depth analysis

For coverage analysis, reads were mapped with BWA-MEM 0.7.4 against the WGS assembly of barley cv. Morex as gene models and exome capture targets are only defined on the Morex assembly [23,25]. Read depth was calculated with ‘samtools depth’ [51]. Regions longer than 150 bp that satisfied one of the following conditions were identified using custom AWK scripts and bedtools [55]: (1) at least 5× average read depth in the wildtype pool and no read coverage in the mutant; (2) the ratio (coverage_mutant/ coverage_wildtype) was at least 4 and the coverage in the mutant pool was ≤2 and ≥5 in the wildtype pool. Condition (2) was chosen to tolerate a small proportion of mis-phenotyped wildtype plants in the mutant pool. The functional annotation of genes located on WGS contigs harboring such regions and the genetic positions of these contigs [23,24] were inspected. Functional annotations were downloaded from [56]. The POPSEQ positions of Morex WGS contigs were retrieved from [57]. The longest putatively deleted region (349 bp) located on a gene-bearing contig (morex_contig_49382 with MLOC_64838.2 annotated as ‘Cytochrome P450’) was assigned to the long arm of chromosome 5H, approximately 95 to 96 cM in the iSelect map [21] and coincided with the peaks of contrasting SNP allele frequency. MLOC_64838.2 was selected as the primary candidate for further validation. Expression data for MND and other CYP78A genes in barley was retrieved from [58].

### Marker development, marker analysis, and genetic mapping

SNPs derived from the exome-capture experiment were converted into CAPS markers (Additional file 3: Table S2) using SNP2CAPS software [59]. Restriction digests were performed according to manufacturer guidelines on a thermocycler. DNA fragments were separated on a 1.5% agrarose gel for genotyping. JoinMap version 4.0 (Kyazma B.V., Wageningen, The Netherlands) with Kosambi mapping function was used to construct a linkage map based on genotyping and phenotypic data.

### PCR amplification and Sanger sequencing

Polymerase chain reaction (PCR) was performed on GeneAmp PCR System 9700 (Applied Biosystems, Carlsbad, CA, USA). A standardized touch down (TD-) PCR profile was used for all PCR analyses containing two cycling steps: initial denaturation for 15 min at 95°C, followed by 10 cycles of denaturation at 95°C / 30 s; annealing at 60°C / 30 s (decreasing by 0.5°C per cycle) followed by extension at 72°C / 60 s); then 35 cycles denaturation at 95°C / 30 s, annealing at 55°C / 30 s, and extension at 72°C / 60 s followed by a final extension step at 72°C / 7 min. PCR products were resolved by agarose gel electrophoresis using 1.5% agarose gel (Invitrogen GmbH, Darmstadt, Germany) strength and 1×TBE buffer. A list of primers used to amplify neighboring genes of MND as inferred by synteny to *B. distachyon* is given in Additional file 3: Table S6.

PCR amplicons were purified with NucleoFast 96 ultra-filtration plates (MACHEREY-NAGEL GmbH & Co. KG, Düren, Germany) and sequenced using BigDye® Terminator v3.1 Ready Reaction Cycle Sequencing Kit (Applied Biosystems, Carlsbad, CA, USA) on the 3730 × l DNA Analyzer (Applied Biosystems, Carlsbad, CA, USA). Obtained sequence reads were analysis was done with ‘Sequencher 4’ software (Genecodes Corporation, USA).

### Identification of mutant alleles

We screened a TILLING population of 10,279 EMS-treated plants of cv. Barke [18] to identify mutant alleles of *HvMND*. Two Primer combinations were used to amplify the full ORF (HvMND_EX1_F/R1 and *HvMND*_Ex2_F/R1; Additional file 3: Table S7) by using PCR with heteroduplex step as described in [18]. PCR products were digested with dsDNA Cleavage Kit and analyzed using Mutation Discovery Kit and Gel - dsDNA reagent kit on the AdvanCETM FS96 system according to manufacturer’s guidelines (Advanced Analytical, IA, USA).

Three oligo combinations (HvMND_F/R1, HvMND_F/R2, HvMND_F/R3) spanning the ORF plus intron were used to resequence the gene in independent *mnd* accessions (Additional file 3: Table S7). Identified SNPs were confirmed by Sanger sequencing (see above). Functional characterization of SNPs was performed using PARSESNP software [60].

### BAC sequencing, assembly, and sequence analysis

A BAC harboring MLOC_64838.2 (HVVMRXALLhB0080C03, FP_contig_45097) was identified by screening a custom re-arrayed BAC library representing all clones of the minimum-tiling path of the genome-wide physical map of barley [29] by amplifying a single gene fragment (HvMND_F/R4, see Additional file 3: Table S7). Contig_46058 was identified as harboring flanking markers based on sequence analysis using available BAC sequences [23]. Thirty-eight BACs from these contigs were shotgun-sequenced on the Illumina HiSeq2000 and assembled with CLC assembly cell version 4.0.6 [61], or on the 454 platform and assembled with MIRA [62]. In addition to MTP clones, we selected additional clones at the ends of FP contigs for sequencing to corroborate potential overlaps between BAC contigs. We also included six previously sequenced BACs [23] in the analysis (Additional file 7: Table S5). Overlap between BACs was detected by an all-against-all alignment with megablast [63] considering only BLAST hits longer than 2 kb and 99.5% sequence identity. BAC sequence contigs were subjected to k-mer-based repeat masking using the Kmasker pipeline [64]. Structural gene annotation of repeat-masked contigs was performed with Augustus [65] using the maize model. Predicted protein sequences were functionally annotated with the AHRD pipeline [66] which parses the description of BLASTP hits against the TAIR [67], Uniprot/trEMBL, and Uniprot/SwissProt [68] databases. Genes annotated as unknown proteins or transposable elements were excluded from further analysis. Gene-bearing Morex WGS contigs were aligned against the BAC assembly with megablast [63] considering only hits longer than 500 bp and a minimum sequence identity of 99.5% to assign IBSC gene models [23] to BACs. Transcript sequences of Augustus models and IBSC genes were clustered with CAP3 [69] to collapse gene models on overlapping BAC clones and to link *ab initio* models to genes in the IBSC annotation.

### Phylogenetic analyses

BLASTP searches [70] against databases of barley [71], *A. thaliana*[72], rice [73], maize, *B. distachyon*[74], *Ae. tauschii*[75], and *T. urartu*[76] proteins were performed to identify CYP78A homologs of MND in these species. A phylogenetic tree was generated with MEGA5 [77] following the protocol of [78]. The evolutionary history was inferred by using the Maximum Likelihood method based on the JTT matrix-based model [79]. The bootstrap consensus tree inferred from 1,000 replicates [80] was taken to represent the evolutionary history of the taxa analyzed. Branches corresponding to partitions reproduced in less than 50% bootstrap replicates were collapsed. Initial trees for the heuristic search were obtained by applying the Neighbor-Joining method to a matrix of pairwise distances estimated using a JTT model. A discrete Gamma distribution was used to model evolutionary rate differences among sites (5 categories (+G, parameter = 1.5089)). The analysis involved 38 amino acid sequences. All positions with less than 80% site coverage were eliminated. That is, fewer than 20% alignment gaps, missing data, and ambiguous bases were allowed at any position. There were a total of 411 positions in the final dataset.

### Data access

Illumina exome sequencing data of two phenotypic pools and BAC sequencing raw data have been deposited at EMBL-ENA as accessions PRJEB5319 (exome capture) and PRJEB5363 (BACs). BAC assemblies are available from GenBank (for accession number see Additional file 7: Table S5). Sanger resequencing data is available at EMBL-ENA (accessions: HG965223 - HG965231).

## Competing interests

The authors declare that they have no competing interests.

## Authors’ contributions

MM performed sequence analysis and drafted the manuscript. MJ performed experiments and helped draft the manuscript. JEK, AH, and AA performed experiments. SB performed BAC assembly. US contributed analysis tools. AG and NS designed research and helped draft the manuscript. All authors read and approved the final manuscript.

## Supplementary Material

Additional file 1: Figure S1 Measurements of plant height, ear length, number of tillers, and number of nodes in 50 wildtype and 50 mutant plants from segregating F_3_ families. Click here for file

Additional file 2: Figure S2 Workflow for exome capture, sequence analysis, and genetic mapping. Click here for file

Additional file 3: Table S2 CAPS markers used for genetic mapping. **Table S6.** Oligonucleotide used to test for complete gene deletions in neighboring genes. **Table S7.** Oligonucleotides used for resequencing and TILLING. Click here for file

Additional file 4: Table S3 List of putative deletions. Click here for file

Additional file 5: Table S1 Detected SNPs with genetic anchoring information and allele frequencies in mutant and wildtype pool. Click here for file

Additional file 6: Table S4 List of resequenced accessions of the Nordic Gene Bank. Click here for file

Additional file 7: Table S5 List of sequenced BACs. Click here for file

Additional file 8: Text S1 AWK script to calculate allele frequency. Usage information is contained within the file. Click here for file
