# Supplementary figures and images for: Mapping-by-sequencing accelerates forward genetics in barley
Source: Genome Biol. 2014 Jun 10;15(6):R78. doi: 10.1186/gb-2014-15-6-r78 (PMC4073093; doi:10.1186/gb-2014-15-6-r78)

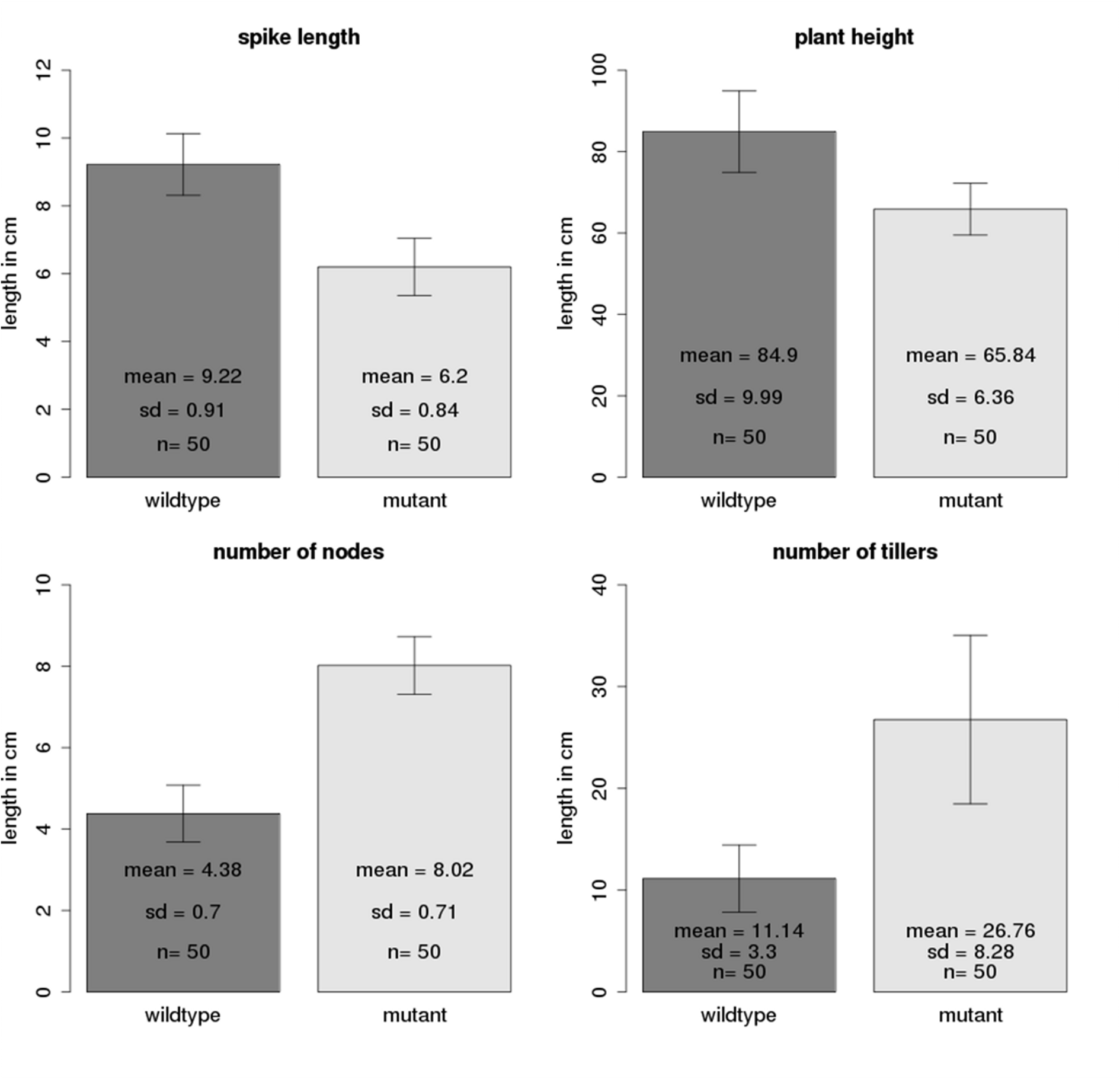

Supplement: Additional file 1: Figure S1 — Measurements of plant height, ear length, number of tillers, and number of nodes in 50 wildtype and 50 mutant plants from segregating F3 families. [file gb-2014-15-6-r78-S1.png]

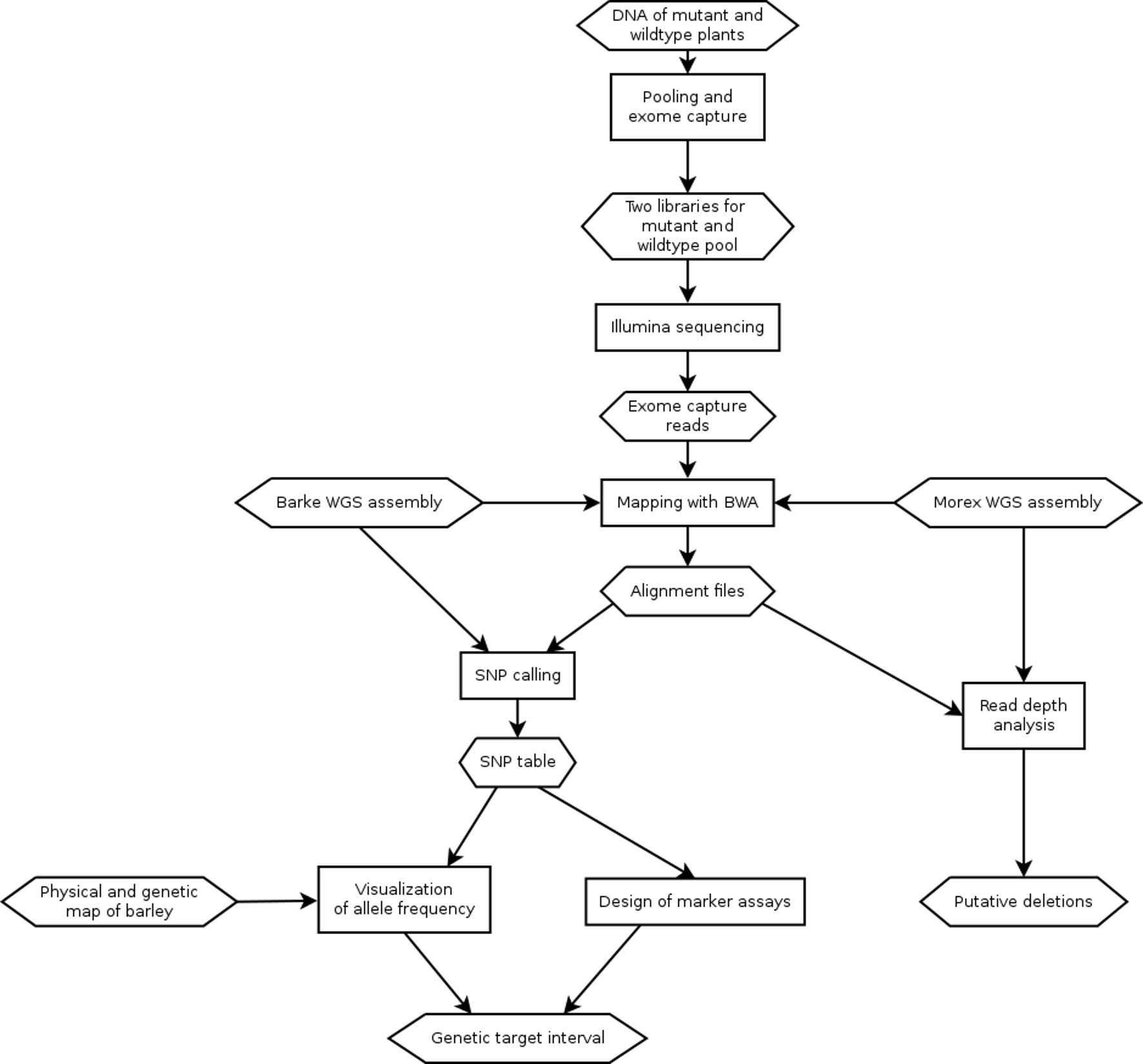

Supplement: Additional file 2: Figure S2 — Workflow for exome capture, sequence analysis, and genetic mapping. [file gb-2014-15-6-r78-S2.png]
