# Supplementary material for: Mapping-by-sequencing accelerates forward genetics in barley
Source: Genome Biol. 2014 Jun 10;15(6):R78. doi: 10.1186/gb-2014-15-6-r78 (PMC4073093; doi:10.1186/gb-2014-15-6-r78)
Supplement: Additional file 3: Table S2 — CAPS markers used for genetic mapping. Table S6. Oligonucleotide used to test for complete gene deletions in neighboring genes. Table S7. Oligonucleotides used for resequencing and TILLING. [file gb-2014-15-6-r78-S3.docx]

**Table S2:** CAPS markers used for genetic mapping.

| **Name** | **Barke WGS contig** | **Forward 5’🡪3’** | **Reverse 5’🡪3’** | **enzyme** |
| --- | --- | --- | --- | --- |
| M1 | contig_1794173 | TCCTGGTCTAGGAGGATGTTCTG | CACTAGCTCCCAGGAGGTACAAA | EcoRI |
| M2 | contig_274977 | AGGATGATCTTCTTGGCCTCCT | GCAGGACAGCTTGAATGTTGAC | HaeII |
| M3 | contig_1781303 | CATTATCCTCTGTTGCCACCTG | CTCGGAGACCCATAAGATCGAG | EcoRI |
| M4* | contig_265820 | CGACGAAGATGACTCTGGAAG | GAACAGCACCACCACCATC | / |
| M5 | contig_1783058 | GATTACGGGGTGGATGCTC | ACCCTCACCTCCAGCCATA | HpyCH4III |
| M6 | contig_267961 | CCACTTCATAGAGTCGCTCCTGT | CGAGAGGATCTATTCGTGTCTGG | HaeII |
| M7 | contig_2786329 | AAGCAGGAGAAGGGTATCAGCTC | GTCAACCGGCAAGCCTTAGATA | HaeII |
| M8 | contig_269740 | GTCTTCCTTCGAGGTGAGCACT | GTGCCGTAGCTGCAACAATAAG | EcoRI |
| M9 | contig_481335 | ATGTATACTGGAGGCTGGAGCTG | AACAGAGCCTGCAAACAAGACC | HindIII |
| M10 | contig_55926 | CTCTCCATCCTCAACTCCTTCCT | GGACCTTGACTATAAGGCTCCAC | HaeII |
|  |  |  |  |  |

* mapped with Sanger sequencing

**Table S6:** Oligonucleotide used to test for complete gene deletions in neighboring genes.

| **Brachypodium gene** | **barley gene** | **FPC contig** | **forward sequence (5' - 3')** | **reverse sequence (5' - 3')** |
| --- | --- | --- | --- | --- |
| [Bradi4g35867.1](http://mips.helmholtz-muenchen.de/plant/brachypodium/reportsjsp/geneticElement.jsp?gene=Bradi4g35867.1) | AK368025 | 45097 | GTCAGGAGCCTCCGTTCC | CTCTCGTCCACCCAGAAGAC |
| Bradi4g35880 | AK355945 | 45097 | CGGCCTCTTCTTCGTCCT | AGGTCACCAGCAAGGTCCTA |
| ***Bradi4g35890.1*** | ***MLOC_64838.2*** | ***45097*** | ***HvMND_F3/R3*** | ***HvMND_F3/R3*** |
| [Bradi4g35900.1](http://mips.helmholtz-muenchen.de/plant/brachypodium/reportsjsp/geneticElement.jsp?gene=Bradi4g35900.1) | MLOC_54310.2 | 46058 | ATCTTCTCCCCCTCCCACT | CTAACATTGGCGCACTTTGA |
| Bradi4g35910 | AK376953 | 46058 | GACGGGGACTGGATGATG | CTCGAGTGGAACGGGAAC |
| [Bradi4g35930.1](http://mips.helmholtz-muenchen.de/plant/brachypodium/reportsjsp/geneticElement.jsp?gene=Bradi4g35930.1) | MLOC_52879.5 | 46058 | GCGCTCTGTGTCCGTCTT | TTTAGTTCACCTCCGGATCG |
| Bradi4g35940 | MLOC_22687.1 | 46058 | GCTCCATCAACGTCTCCTTC | GCAAGACGTCCAAGAAGTCC |
| [Bradi4g35950.1](http://mips.helmholtz-muenchen.de/plant/brachypodium/reportsjsp/geneticElement.jsp?gene=Bradi4g35950.1) | AK374133 | 46058 | AAGCTTGCTAGTCGGCATGT | GTGGTTCACCATTTGCTCCT |
|  |  |  |  |  |

**Table S7:** Oligonucleotides used for resequencing and TILLING.

| **Name** | **forward sequence (5' - 3')** | **reverse sequence (5' - 3')** |
| --- | --- | --- |
| HvMND_F1/R1 | CCCTACTCCTCGATGACGAT | ACACGGACCACATCACGTT |
| HvMND_F2/R2 | CACCATCTCCTGCAACACAC | CCAGGGAGATCCTCAACAGC |
| HvMND_F3/R3 | CATCGCTTGCTAGTTGCTCA | ACATGATCGCGGTGCTCT |
| HvMND_F4/R4 | GTGCGTGCTTGCTTGCTA | GTGCTCTGGGTGTGTGCAA |
| HvMND_EX1_F1/R1 | GCCTTTTCCTGCGGCTATAA | CAGAGAATACCCCCAACGAA |
| HvMND_EX2_F2/R2 | GTCCTGCTCTCCCTCCAAG | CGCTTGCTAGTTGCTCATCA |
